# Effects of social participation patterns and living arrangement on mental health of Chinese older adults: A latent class analysis

**DOI:** 10.3389/fpubh.2022.915541

**Published:** 2022-08-05

**Authors:** Jieyao Chen, Yanbing Zeng, Ya Fang

**Affiliations:** ^1^Key Laboratory of Health Technology Assessment of Fujian Province, School of Public Health, Xiamen University, Xiamen, China; ^2^School of Public Health, Capital Medical University, Beijing, China

**Keywords:** older adults, social participation, latent class analysis, mental health, living arrangement

## Abstract

**Objectives:**

The purpose of this study was to discover patterns of social participation among Chinese older individuals, investigate the links between these patterns and their living arrangements and mental health, and connect these patterns to their background characteristics.

**Methods:**

Data were drawn from the 2014 and 2018 waves of Chinese Longitudinal Healthy Longevity Survey (CLHLS) from 2537 Chinese elders aged 60 years and over at the baseline. Latent class analysis was used to determine the patterns of social participation. Multinomial logistic regression was used to explore the relationship between patterns of social participation and the sociodemographic and health characteristics of older adults. Binary logistic regression was used to explore the differences and associations between these categories and living arrangement on mental health status, represented by positive and negative emotions.

**Results:**

Three patterns of social participation were identified: Low activity (17.5%, *n* = 443); Moderate activity (36.2%, *n* = 1,176); and High activity (46.3%, *n* = 918). At baseline, these patterns were significantly associated with mental health status. Positive and negative emotions were much better in the High activity group than in the other two groups (OR = 1.36, 95% CI = 1.05–1.76 and OR = 1.50, 95% CI = 1.16–1.93). Living arrangement only significantly affected negative emotions (OR=1.25, 95% CI = 1.02–1.53). Age, gender, education, marital status, self-rated health, and activities of daily living limitations all affected social participation patterns.

**Conclusions:**

The patterns of social participation and living arrangement of Chinese older adults are significantly associated with mental health. Population-tailored interventions may help liberate older adults from domestic labor and improve social participation. Moreover, family support can decrease negative emotions' damage in old age thus promoting health.

## Background

The World Health Organization (WHO) defines health as a state of complete physical, mental, and social wellbeing, not merely the absence of disease or frailty. It is evident that mental health is an important component of health. However, as older adults age, they inevitably develop negative emotions such as depression, anxiety, and loneliness due to physical frailty and cognitive decline. As social changes have accelerated, more and more older adults around the world are suffering from negative emotions (1). These may lead to poor coping and increased risk of chronic disease and mortality (2). On the contrary, positive emotions not only mark current wellbeing, but also contribute to future wellbeing (3). Previous studies have shown that positive emotions have been linked to slower physical degeneration, better physical functioning, and increased longevity (4). The older people's mental health has a direct impact on their attitude toward life and satisfaction, but it also has an indirect impact on their physical health, influencing their quality of life later in life. As a result, it is critical to look into the mental health status of the older people, which comprises both positive and negative emotions, in order to enhance the quality of life and promote social harmony.

The cognitive-motivational-relational theory of emotion holds that positive and negative emotions are always caused by external factors and, as an external factor, families have a deep and long-lasting impact on an array of older adults' health outcomes (1, 5). The mental health status of older adults in China is directly linked to their families. Even though ongoing changes in social structure such as further reduction in the number of household members may diminish a traditional family's responsibilities, family remains a significant source of support for older adults and constitutes a living and emotional anchor for many Chinese seniors. Previous research has found that a person's living arrangement has a significant impact on their physical and mental health (6), with older Vietnamese and Thai adults living with family members being more likely to receive help and companionship and maintain good physical and mental health. Conversely, older adults living alone are more likely to experience negative emotions due to increased loneliness (7). Therefore, the significance of living arrangements in the aging process should be highlighted.

Social participation is defined as an individual's activity in real life, and it plays an important role in reinforcing social relationships, social support, and social integration, resulting in better health and related outcomes. It is an important component of active aging, and it is a central topic in many aging studies (8–10). Social participation not only helps older adults maintain social connections after retirement and avoid social isolation, but it also improves physical and mental health by allowing them to reap the benefits of communication and interaction with others, as well as promoting their physical activity. Social participation has been shown in studies to lower the risk of depression and cognitive impairment, and improve aspects like self-rated health, wellbeing, life satisfaction, quality of life, and even older adults' ability to control negative emotions and maintain a positive sense of self and worth (11–13). Further, depending on the type of activity, gender, and place of residence, the association between social participation and health may differ (14). There is now widespread agreement that social participation is essential for active, healthy aging, and it is important to investigate its impact on older adults' mental health.

Drawing on the above literature, most studies focused on all types of social participation without distinction between them. But many correlated interrelationships exist between measures of social participation and activities and these are not well understood (15). Therefore, a statistical technique like latent class analysis (LCA) seemed more appropriate because it takes these interrelationships into account (16). A further advantage of LCA is that it groups individuals rather than activities (i.e., person-centered vs. item-centered) (17), this may provide new ideas for identifying the relationship between population type and risk for mental health or even the exploration of mechanisms. Most previous studies only used depression and anxiety as sole indicators of mental health status, failing to account for the diversity of emotions experienced by older adults and providing a more comprehensive picture of mental health. Therefore, this study employed national survey data to investigate the latent types of social participation in China, as well as the relationship between these types of social participation, living arrangements, and the mental health status of older adults. In addition, we looked into the impact of older adults' individual characteristics on different types of social participation in an attempt to develop a multilevel and comprehensive analytical framework.

## Methods

### Data and sample

The data were derived from the 2014 and 2018 waves of Chinese Longitudinal Healthy Longevity Survey (CLHLS), which is part of a collaborative project between Duke University in the United States and Peking University in China. It aims to better understand the health status of elderly Chinese people and the social, behavioral, and biological factors that influence their health (12). CLHLS was provided to a sample of 22 out of 31 provinces in China. This survey was conducted in the years 1998, 2000, 2002, 2005, 2008, 2011, 2014, and 2018. We chose 2014 as the baseline year to acquire complete baseline data on the relevant covariates, such as social participation and living arrangement, and 2018 as the follow-up year to create a four-year cohort. This provided a total baseline sample of *n* = 7,192 individuals in 2014. However, we excluded *n* = 1329 individuals with missing social participation key variables, *n* = 32 individuals aged younger than 60, and *n* = 3294 individuals who died before 2018. Thus, the remaining sample consisted of *n* = 2537 respondents for further analyses.

### Measures

#### Social participation and living arrangement

On the basis of previous similar studies (18–20), a total of eight questions were asked as baseline measure of social participation. These explored engagements included the following activities: housework, fieldwork, gardening, reading newspapers/books, raising pets, playing cards/mah-jongg, watching TV or listening to the radio, and taking part in any social activities. Their answers were dichotomized into “yes” (=1) or “no” (=0). Similarly, living arrangement was divided into living alone (=0) or living with family (=1).

#### Mental health

Positive and negative emotions were used as proxy variables to measure the mental health status of older adults. Positive emotions were selected from the CLHLS questionnaire, including: “self-reported quality of life”, “look on the bright side of things” and “do you feel as happy as you were when you were young”. Negative emotions included “are you nervous and scared”, “do you feel lonely”, and “do you think that the older you become, the less useful you are”. Each of these entries takes a value between 1–5, with 1 indicating “very good” or “always”, and 5 indicating “very bad” or “never”. The entries were summed and the mean value was calculated. Any value below the mean represents stronger positive emotions or negative emotions in older adults (higher positive emotions/lower negative emotions = 1). Values greater than, or equal to, the mean represent weaker positive emotions or negative emotions in older adults (lower positive emotions/higher negative emotions = 0).

#### Covariates

Potentially relevant covariates at baseline were obtained from sociodemographic characteristics, health, and economic status. All variables were dichotomized. For sociodemographic characteristics, age was measured in chronological years. In this study, 60–84 year-olds were considered “low age” (0), elders aged 85 and over were considered “high age” (1). Gender was dichotomized as “men” (1) or “women” (0). Residence was dichotomized as “city/town” (1) or “rural” (0). Education was divided into “illiterate” (0) and “non-illiterate” (1), depends on formal schooling or not. Similar to previous CLHLS studies, occupational status was defined as “high level” (1) if the participant's primary occupation before age 60 was professional, technical, governmental, institutional, managerial, or military (21). Marital status was categorized as “married” (1) or not (0). Income was categorized as above (1) or below (0) the average income of the entire sample. For self-rated health, we regard the “good/very good” answer as “good” (1) or not (0). As for the health status, activities of daily living (ADL) were referred to as basic activities of daily living (BADL). BADL includes six daily tasks: dressing, eating, toileting, bathing, indoor activities, and continence. Disability in BADL was defined as an inability to perform any of the corresponding tasks independently (0). The multiple imputation approach was applied to reduce the influence of missing values on these covariates in the analyses (22).

### Statistical analysis

LCA is a statistical classification process based on the probability of detecting unobserved population heterogeneity in response patterns. With LCA, an unordered categorical latent factor with a multinomial distribution is responsible for the link between observed (categorical) indicators, resulting in homogenous subtypes or mixtures. The resulting mixes (latent classes) are qualitatively distinct, with members within a class differing primarily in terms of random measurement error. However, based on their pattern of response profiles, they are distinctively and systematically different from members in other classes (23). The goal of LCA is to find the fewest unobserved classes that sufficiently account for observed relationships between symptoms in the data, and place the individuals with similar symptom profiles in the same class. The most parsimonious class model is fitted to the data, followed by subsequent models with increasing class numbers to discover the optimal number of latent classes (24). In this analysis, latent classes were identified based on 8 dichotomous indicators of social participation of CLHLS. Model fit was assessed by the Akaike Information Criterion (AIC), Bayesian Information Criterion (BIC), sample size adjusted BIC (aBIC), entropy, the Vuong-Lo-Mendell-Rubin likelihood ratio (LMR) test, and the bootstrapped likelihood ratio tests (BLRT) (25).

To summarize the characteristics and differences of the entire sample against each social participation class, we employed frequencies, proportions, and Chi-square tests. In our LCA model, binary logistic models were used to test the relationship between latent social participation classes and living arrangement with positive and negative emotions. Relative risk ratios (95% confidence interval) were used to describe the association between them. All models were adjusted for age, sex, residence, education, income, occupation, marital status, self-rated health, and ADL limitations.

Multinomial logistic models were used to identify factors associated with social participation patterns of Chinese elders. LCA models and analysis were conducted on MPlus (v8.0), and all subsequent analyses were performed on R (v4.1.0).

## Results

### Patterns of social participation: A three-class model

**Figure 1:**
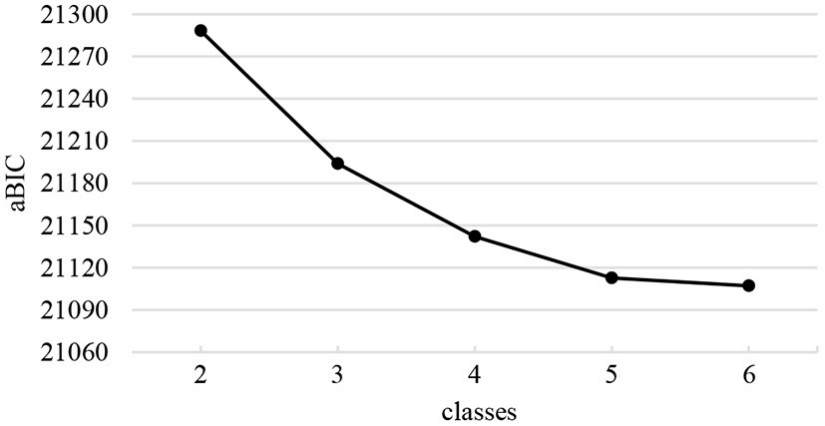
Steep slope diagram test of the LCA model.

**Figure 2:**
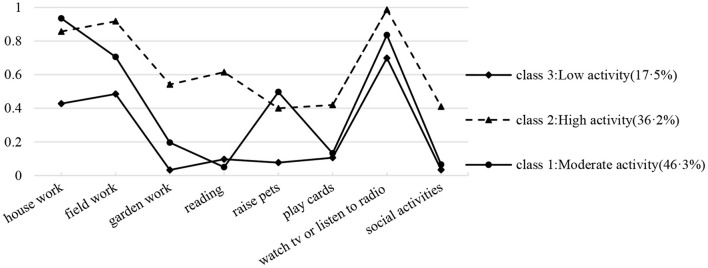
Conditional probability distribution for three categories of social participation (*N* = 2,537).

Table 1 shows the LCA results. Five latent class models were created in this study, combining the metrics and considering interpretability. The simulation study found that the sample-size adjusted BIC (aBIC) is the information index with the highest classification accuracy, though aBIC will continue to decrease as potential classes increase. Therefore, this study used a steep slope diagram test (Figure 1), and it can be seen that the most obvious inflection point exists at class 3. Meanwhile, the higher entropy value represents the stronger classification accuracy. Although entropy is best for the five-class model (0.60), the steep slope diagram test does not support this as the best model. The three-class model has the second-highest entropy value (0.586). Combining the LMR index, steep slope diagram, and interpretability (Figure 2), made the three-class model the best option.

**Table 1:** LCA model fit statistics (*N* = 2,537).

**Classes**	**AIC**	**BIC**	**aBIC**	**Entropy**	**LMR**	**BLRT**
2	21243.004	21342.262	21288.249	0.549	<0.0001	<0.0001
3	21124.698	21276.505	21193.896	0.586	0.0014	<0.0001
4	21048.975	21253.331	21142.126	0.536	0.1522	<0.0001
5	20995.624	21252.529	21112.729	0.600	0.0089	<0.0001
6	20966.045	21275.498	21107.103	0.586	0.1229	<0.0001

AIC, Akaike information criteria; BIC, Bayesian information criteria; aBIC, sample-size adjusted BIC; LMR, p-value for the Lo-Mendell-Rubin likelihood ratio test.

Class 3 (17.5%) had low probabilities of engaging in social participation across eight social participation factor domains. We therefore labeled class 3 as “Low activity.” Because these groups engaged in each activity domain at a “medium” and “high” level, we labeled classes 1 and 2 as “Moderate activity” (46.3%) and “High activity” (36.2%), respectively. Furthermore, class 1 demonstrated a significant preference for a type of participation (Figure 2) with a focus on home-based activities, such as housework and watching TV.

### Participant characteristics

Table 2 reflects the demographic characteristics of the participants. In this study, 1,877 older adults (74.0%) were under the age of 85 years, with slightly more men (*n* = 1,300) than women (*n* = 1,237). About half of the elderly live in the countryside (56.5%), have no education (54.1%), have above-average income (54.3%), and are married (55.5%).The vast majority of older adults had a low occupational status prior to retirement (95.3%) and lived with their families (78.3%). In terms of health status, almost equal numbers of older adults rated themselves in good (50.3%) and poor (49.7%)health, with the vast majority (93.5%) having good activities of daily living.

**Table 2:** Participants characteristics and group differences among three patterns of social participation at the baseline (*N* = 2,537).

**Characteristics**	**Whole sample** **(*n =* 2,537)** **n (%)**	**Class 1** **(*n =* 1,176)** **n (%)**	**Class 2** **(*n =* 918)** **n (%)**	**Class 3** **(*n =* 443)** **n (%)**	***P*** **value**
Age group (years)					<0.001
60–84	1,877 (74.0)	885 (75.3)	765 (83.3)	227 (51.2)	
≥ 85	660 (26.0)	291 (24.7)	153 (16.7)	216 (48.8)	
Gender					<0.001
Male	1,300 (51.2)	465 (39.5)	614 (66.9)	221 (49.9)	
Female	1,237 (48.8)	711 (60.5)	304 (33.1)	222 (50.1)	
Residence					<0.001
City or town	1,104 (43.5)	433 (36.8)	482 (52.5)	189 (42.7)	
Rural	1433 (56.5)	743 (63.2)	436 (47.5)	254 (57.3)	
Education					<0.001
Non-illiterate	1,372 (54.1)	482 (41.0)	703 (76.6)	187 (42.2)	
Illiterate	1,165 (45.9)	694 (59.0)	215 (23.4)	256 (57.8)	
Occupation					<0.001
High level	119 (4.7)	8 (0.7)	10 (2.3)	101 (22.8)	
Low level	2,418 (95.3)	1,168 (99.3)	908 (97.7)	342 (77.2)	
Marital status					<0.001
Married	1,409 (55.5)	605 (51.4)	633 (69.0)	171 (38.6)	
Others	1,128 (44.5)	571 (45.6)	285 (31.0)	272 (61.4)	
Income (Yuan)					<0.001
Above average income	1,377 (54.3)	543 (46.2)	596 (64.9)	238 (53.7)	
Below average income	1,160 (45.7)	633 (53.8)	322 (35.1)	205 (46.3)	
Living arrangements					<0.001
Living with family	1,987 (78.3)	887 (75.4)	764 (83.2)	336 (75.8)	
Living alone	550 (21.7)	289 (24.6)	154 (16.8)	107 (24.2)	
Self-rated health					<0.001
Very good, good	1,277 (50.3)	546 (46.4)	542 (59.0)	189 (42.7)	
Fair, poor, or very poor	1,260 (49.7)	630 (53.6)	376 (41.0)	254 (57.3)	
ADL limitations					<0.001
No	2,371 (93.5)	1,127 (95.8)	883 (96.2)	361 (81.5)	
Yes	166 (6.5)	49 (4.2)	35 (3.8)	82 (18.5)	
Positive emotions in 2018				<0.001
Higher	1,058 (41.7)	446 (37.9)	456 (49.7)	156 (35.2)	
Lower	1,479 (58.3)	730 (62.1)	462 (50.3)	287 (64.8)	
Negative emotions in 2018					<0.001
Lower	1,419 (55.9)	612 (52.0)	595 (64.8)	212 (47.9)	
Higher	1,118 (44.1)	564 (48.0)	323 (35.2)	231 (52.1)	

In addition, Table 2 shows the baseline sample characteristics and group differences among three patterns of social participation. In terms of mental health, more than half of the people with lower positive emotions (58.3%, *n* = 1,479) and lower negative emotions (55.9%, *n* = 1,419). Significant differences were found in the three patterns of social participation among the groups in all characteristics.

### Factors affecting the patterns of social participation: A multi-category logistic regression

The multinomial logistic regression model was used to identify the predictors of each social participation class (Table 3). Respondents who were ≥85 years old (OR=0.39, 95%CI = 0.30–0.50, OR = 0.38, 95% CI = 0.28–0.50, respectively) were less likely to be in the “Moderate activity” or “High activity” groups than in the “Low activity” group. Likewise, respondents who were married (OR=1.71, 95%CI = 1.29–2.28, OR = 2.37, 95% CI = 1.73–3.24, respectively), without ADL limitations (OR = 4.75, 95%CI = 3.20–7.04, OR = 4.93, 95%CI = 3.09–7.84, respectively) were more likely to be in the “Moderate activity” or “High activity” groups than in the “Low activity” group. Furthermore, respondents who were male (OR = 0.47, 95%CI = 0.36–0.61) and lived with family (OR = 0.67, 95%CI = 0.47–0.95) were less likely to be in the “Moderate activity” group or “High activity” group, respectively. Respondents who had good self-rated health (OR = 1.91, 95% CI = 1.48–2.46), and a higher education level (OR = 2.70, 95%CI = 2.02–3.60) were more likely to be in the “High activity” group than in the Low activity group.

**Table 3:** Multinomial logistic regression for social participant class.

**Variables**	**Social participation class** **(ref.: Low activity class)**					
	**Class 1: Moderate activity (*****n** =* **1,176)**			**Class 2: High activity (*****n** =* **918)**		
	**OR**	**95%CI**	**P**	**OR**	**95%CI**	**P**
≥ 85	0.39	0.30-0.50	<0.001	0.38	0.28-0.50	<0.001
(ref. 60-84)						
Male	0.47	0.36-0.61	<0.001	0.88	0.66-1.18	0.40
(ref. female)						
Living in city/town	0.87	0.68-1.11	0.27	1.24	0.95-1.62	0.12
(ref. rural)						
Education	0.99	0.76-1.29	0.93	2.70	2.02-3.60	<0.001
(ref. illiteracy)						
Occupation	0.38	0.14-1.03	0.06	1.81	0.87-3.79	0.11
(ref. low level)						
Married	1.71	1.29-2.28	<0.001	2.37	1.73-3.24	<0.001
(ref. others)						
Income	0.86	0.67-1.12	0.28	1.23	0.92-1.63	0.16
(ref. ≤ average)						
Living with family	0.84	0.61-1.15	0.27	0.67	0.47-0.95	0.02
(ref. living alone)						
Self-rated health	1.20	0.95-1.51	0.13	1.91	1.48-2.46	<0.001
(ref. bad)						
Without ADL limitations	4.75	3.20-7.04	<0.001	4.93	3.09-7.84	<0.001
(ref. yes)						

CI, confidence interval; OR, odds ratio; ref, reference.

### Relationship between living arrangement, patterns of social participation, and mental health

Table 2 shows that baseline patterns of social participation were significantly associated with positive and negative emotions in older adults in 2018. The probability of having higher positive emotions was 35.2, 37.9, and 49.7% for those in the Low activity group, Moderate activity group, and High activity group, respectively, and for having lower negative emotions these were 47.9, 52.0, and 64.8% (χ*2* test, *p* < 0.001). Positive and negative emotions were much better in the High activity group than in the other two groups. Further, the probability of higher levels of positive emotions and lower levels of negative emotions both gradually decreased as the level of participation increased.

After correcting for sociodemographic and health-related characteristics, patterns of social participation were significantly associated with both positive and negative emotions among older adults in binary logistic regression models (Tables 4, 5), whereas living arrangement only significantly affected negative emotions. “High activity” elders had higher positive emotions and lower negative emotions than “Low activity” counterparts (OR = 1.36, 95%CI = 1.05–1.76 and OR = 1.50, 95%CI = 1.16–1.93). Meanwhile, “Low activity” and “Moderate activity” older adults had no statistically significant differences in both positive and negative emotions. In terms of living arrangement, living with family members or not had no significant effect on their level of positive emotion. Conversely, the level of negative emotion was much lower in older adults living with family members than in older adults living alone.

**Table 4:** Logistic regression models between living arrangement, patterns of social participation and positive emotions (*n* = 2,537).

	**Model 1**	**Model 2**	**Model 3**
**Variable**	**OR (95% CI)**	**OR (95% CI)**	**OR (95% CI)**
Living arrangement	0.98 (0.80–1.22)		0.99 (0.80–1.22)
**Patterns of SP (Low activity as reference)**			
High activity		1.36^*^ (1.05–1.76)	1.36^*^ (1.05–1.76)
Moderate activity		1.09 (0.86–1.39)	1.09 (0.86–1.39)

All models were adjusted for age, gender, residence, education, income, occupation, marital status, self-rated health, and ADL limitations.

* p < 0.05.

Model 1: association between only living arrangement and positive emotions.

Model 2: association between only patterns of social participation and positive emotions.

Model 3: association between both living arrangement, patterns of social participation and positive emotion.

**Table 5:** Logistic regression models between living arrangement, patterns of social participation and negative emotions (*n* = 2,537).

	**Model 1**	**Model 2**	**Model 3**
**Variable**	**OR (95% CI)**	**OR (95% CI)**	**OR (95% CI)**
Living arrangement	1.25^*^ (1.02–1.53)		1.25^*^ (1.02–1.53)
**Patterns of SP (Low activity as reference)**			
High activity		1.50^**^ (1.16–1.93)	1.50^**^ (1.16–1.93)
Moderate activity		1.14 (0.90–1.44)	1.14 (0.90–1.44)

All models were adjusted for age, gender, residence, education, income, occupation, marital status, self-rated health, and ADL limitations.

* p < 0.05;

** p < 0.01.

Model 1: association between only living arrangement and negative emotions.

Model 2: association between only patterns of social participation and negative emotions.

Model 3: association between both living arrangement, patterns of social participation and negative emotions.

## Discussion

By applying clustering method on a nationally representative sample of elders to investigate their patterns of social participation and factors influencing their classification, we discovered that positive and negative emotions were associated with patterns of social participation, living arrangements, and mental health in older adults. We also explored the social individual characteristics and health issues that may influence participation patterns and preferences across different populations.

Our study identified three patterns of social participation among elders in China: “Low activity”, “Moderate activity”, and “High activity”. This is similar to Chen and colleagues' study. Three classes differed among several aspects of social participation. A main difference regarded the level of activity in social participation. Further, the “Moderate activity” group, in addition to its intensity, reflects essential aspects of traditional leisure such as housework, fieldwork, watching tv, and listening to the radio, and the number of elders in this category is the highest of the three. This outcome matches traditional Chinese culture and other scholars' studies (26). Many Chinese seniors devote most of their time and energy to their families. The extent of their conspicuous turnout in the family is attributable to family responsibilities, otherwise forced choice is an issue that warrants further investigation. Moreover, the most noticeable difference between the “High activity” group and the other two is the level of participation in self-help activities such as reading, playing cards, and other social activities. This is possibly the main reason why the three social participation patterns exhibit distinct mental health benefits.

Our findings show a significant association between patterns of social participation and mental health among older adults. We found that the elders in the “High activity” group were most likely to engage in all eight activities. They also reported significantly lower negative emotions and significantly higher positive emotions. These results corroborate findings reported in another study (27) where older adults who participate in a variety of social activities may exhibit better mental health status. Only at the highest level of social participation (“High activity” group) compared to the “Low activity” group, both positive and negative emotions of the older people were better. Similar studies have demonstrated that social activity may increase social interaction and enhance feelings of enjoyment, self-worth, and self-esteem, and the effect is regulated by their intensity (28). This further validates previous research (29) stating that not all social participation contributes to the mental health of older adults. Social participation has a relatively limited effect on both positive and negative emotions in older adults who are in the “Moderate activity” (domestic types) and “Low activity” levels. The “High activity” group, in particular, has the highest level of social participation, and the older people themselves are the primary beneficiaries of the related activities. “Moderate activity” and “Low activity” groups are mostly focused on family and traditional recreation, which do not require strong physical strength, intelligence, or socializing among peers. The beneficiaries are more the families than themselves, so the influence on the elderly's mental health is not significant. In general, our results on the association between social participation and mental health are in line with previous studies (30, 31).

It is worth mentioning that living with families shields older persons from negative emotions, but has no influence on positive ones. Due to cognitive decline, physical health decline, and traumatic life events such as the loss of a spouse and property, older adults are more sensitive to low mood. In western elders, friend networks have a stronger impact on mental health than family (32). “Raising sons for old age” is a well-known Chinese saying. For Chinese elders, we speculate that, living with families can increase the experience of intimacy, life satisfaction, and psychological needs, limiting negative emotions like loneliness and depression. However, the enhancement of positive emotions should rely on elders actively participating in social activities that benefit them, to gain a sense of value and pleasure. Older Chinese people devote most of their retirement time to caring for their grandchildren in spite of other forms of socialization (33). Therefore, families should assist and encourage older adults to engage in activities and integrate into society, as well as prove financial and emotional support. Strengthening intergenerational communication and reaffirming the role of family in elders' health promotion can help mitigate and prevent physical and mental harm caused by negative emotional shocks, while encouraging the elderly to age with a partner and live with their children for more emotional support. Lifecare can help improve their health status.

Age, gender, education, marital status, living arrangement, self-rated health, and ADL limitations were all found to influence the type of social participation in our study. Male aged 85 and over had a higher probability of being in the “Low activity” group. In contrast, older persons who are educated, married, in good self-rated health, and disability-free are more likely to be in the “High activity” group. Females are more enthusiastic about social participation than males, presumably because they have a stronger desire for emotional support or involvement in group or community activities (34). Furthermore, these more involved older persons were more likely to be living alone, as also found elsewhere (35). The association between educational attainment and social participation is also consistent with previous studies. Older adults with higher levels of education are more likely to reach a higher level of social participation. This not only highlights education as a coping resource but also demonstrates that socioeconomic status in general affects older adults' social activities. Older adults with poor self-rated health, or unable to care for themselves fully were more likely to engage in static, less intensive, and limited entertainments activities.

### Strengths and limitations

This study makes some contributions to the current, relevant, body of evidence. First, to the best of our knowledge, this is the first study that applies LCA to nationally representative data to identify patterns of social participation among Chinese elders, whereas previous studies have typically classified social participation directly by activity name. This circumvents the problem that different social participation patterns may be too highly correlated to be compared directly. Second, our research used positive and negative emotions as measures of mental health status, whereas previous studies have used single indicators like depression, anxiety, and wellbeing as evaluation criteria. Finally, considering that the results of cross-sectional studies likely overestimate the association between social participation and health and wellbeing due to (time-constant) unobserved confounders (29), our use of longitudinal data from 2014 to 2018 to conduct a preliminary exploration of the causal relationship between types of social participation and mental health provides a novel solution to this problem.

It must be acknowledged that this study does suffer from some limitations. This study did not use multiple waves of data to examine the dynamic patterns of social participation. Future studies could encompass multiple waves of data in social activities among older adults. This study used only eight indicators of social participation. Since social participation encompasses not only social activities but also social support, and that good interpersonal relationships could indirectly and positively influence older adults' mental wellbeing (36), it would be more useful to incorporate social networks in the scope of social participation thus broadening the scope of the assessment.

## Conclusions

The three social participation patterns discovered in this study differed in terms of participation intensity and preference type. They reflected diverse gender, age, educational level, and health-related variables.

This research lays a solid foundation for boosting older adults' social integration, improving their mental health, and encouraging healthy and active aging among the older people. When compared to previous research, this study adds to the understanding of the link between different types of social participation, living arrangements, and mental health as expressed by positive and negative emotions in older adults. Therefore, families, society and government can identify mental health risks in advance through the heterogeneous personal characteristics of older adults such as social participation characteristics, age and marriage, and tailor effective interventions for different groups of people, so as to improve older adults' mental health by encouraging them to participate in social activities within their means, thereby maximizing the elimination of negative emotions generated during the aging process and obtaining more positive emotions.

## Data availability statement

The datasets presented in this study can be found in online repositories. The names of the repository/repositories and accession number(s) can be found below: The datasets analyzed during the current study was publicly available at http://chads.nsd.pku.edu.cn/sjzx/index.htm.

## Ethics statement

The studies involving human participants were reviewed and approved by Duke University Health System's Institutional Review Board (Ethics Number: IRB00001052-13074). The patients/participants provided their written informed consent to participate in this study.

## Author contributions

YZ conceived and designed the study and supervised the data analysis. YZ and JC wrote the paper. JC performed all statistical analyses. YZ and YF contributed to revising the paper. All authors contributed to the article and approved the submitted version.

## Funding

This study was funded by National Natural Science Foundation of China (Nos. 71874147 and 81973144). The funder had no role in the study design, the collection, analysis and interpretation of the data, the writing of the report, and the decision to submit the article for publication.

## Conflict of interest

The authors declare that the research was conducted in the absence of any commercial or financial relationships that could be construed as a potential conflict of interest.

## Publisher's note

All claims expressed in this article are solely those of the authors and do not necessarily represent those of their affiliated organizations, or those of the publisher, the editors and the reviewers. Any product that may be evaluated in this article, or claim that may be made by its manufacturer, is not guaranteed or endorsed by the publisher.
